# Regenerative cerium oxide nanozymes alleviate oxidative stress for efficient dry eye disease treatment

**DOI:** 10.1093/rb/rbac070

**Published:** 2022-09-21

**Authors:** Haoyu Zou, Haiting Wang, Baoqi Xu, Lin Liang, Liangliang Shen, Quankui Lin

**Affiliations:** Department of Biomaterials, School of Ophthalmology & Optometry, Eye Hospital, Wenzhou Medical University, Wenzhou 325027, P. R. China; Department of Biomaterials, School of Ophthalmology & Optometry, Eye Hospital, Wenzhou Medical University, Wenzhou 325027, P. R. China; Department of Biomaterials, School of Ophthalmology & Optometry, Eye Hospital, Wenzhou Medical University, Wenzhou 325027, P. R. China; Department of Biomaterials, School of Ophthalmology & Optometry, Eye Hospital, Wenzhou Medical University, Wenzhou 325027, P. R. China; Department of Biomaterials, School of Ophthalmology & Optometry, Eye Hospital, Wenzhou Medical University, Wenzhou 325027, P. R. China; Department of Biomaterials, School of Ophthalmology & Optometry, Eye Hospital, Wenzhou Medical University, Wenzhou 325027, P. R. China

**Keywords:** cerium oxide nanozymes, dry eye, reactive oxygen species, oxidative stress

## Abstract

Dry eye disease (DED) is the most common eye disease in ophthalmic consultation except for refractive errors. Therefore, an exploration of valid and alternative therapeutic interventions is essential to feed the urgent medical need. It has been demonstrated that oxidative stress causes multiple adverse effects in the pathogenesis of DED, thence alleviating oxidative stress is an effective therapeutic strategy for the DED treatment. Herein, we developed a cerium oxide nanozyme combined with branched poly(ethylene imine)-graft-poly(ethylene glycol) (bPEI-g-PEG). Owing to its stable hydrophilic chains on the surface reducing the cytotoxicity and loads of amines groups that be combined with cerium ions through coordination bonds, the modified nanozymes (referred to as CNP@bPEI-g-PEG) are water soluble and highly biocompatible. Meanwhile, due to its excellent antioxidant activity, CNP@bPEI-g-PEG nanozymes can mimic the activity of superoxide dismutase and catalase to scavenge intracellular reactive oxygen species (ROS). Experimental studies firmly demonstrated that the modified nanozymes were auto-regenerative and more active in scavenging excessive ROS and alleviating oxidative stress by cerium-element valence state recycling, recovering the morphology of corneal, conjunctival epithelium and the number of goblet cells. The advanced combination may offer a superior therapeutic strategy to deal with oxidative stress for effective treatment of DED.

## Graphical Abstract

**Figure:**
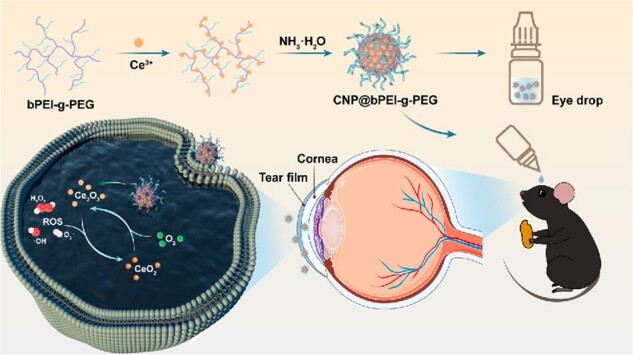

## Introduction

Dry eye disease (DED), also known as keratoconjunctivitis sicca, is a multifactorial disease that causes damage to the tear film and ocular surface tissue, resulting in various ocular discomforts such as eye fatigue, foreign body sensation, dryness, irritation, visual impairment, etc. [1]. DED is the most common eye disease in ophthalmic consultation except for refractive errors [2]. The prevalence of DED is surging dramatically, with and without symptoms, ranging from 5% to 50% in the world [3, 4], disrupting seriously visual-life quality and imposing billions of economic burdens on society. In recent years, with the high-frequency use of video display terminals (mainly computers, mobile phones, etc.), the increase of corneal contact lens users, refractive surgery and various environmental factors, the prevalence of DED has shown an upward and younger trend [5]. DED affects the quality of life seriously, and the prevalence of anxiety and depression among DED patients is obviously higher than that in healthy people [6]. At present, DED treatments including artificial tears, anti-inflammatory drugs, physical therapy and surgery mainly focus on controlling the symptoms and relieving ocular discomforts, but the effect is not ideal, and the recurrence rate is extremely high [7, 8]. The treatment is more difficult especially for patients with severe DED symptoms. Therefore, focusing on the cause is the key and fundamental means of curing DED.

Although the pathogenesis of DED is not fully clarified, tear hyperpermeability and immune inflammatory response have been recognized as a hallmark of the disorder [9–11]. However, oxidative stress caused by reactive oxygen species (ROS) imbalance plays a decisive role in the progress of DED [12]. ROS, such as superoxide anion radical ($O_{2}^{-\cdot }$), hydrogen peroxide (H_2_O_2_), hydroxyl radical (OH·) and singlet oxygen, are by-products of aerobic metabolism in mitochondria [13, 14]. Under normal physiological circumstances, ROS plays an essential role in the redox regulation of mitochondrial oxidative phosphorylation. Furthermore, ROS are balanced by biological antioxidant enzyme systems. When ROS production exceeds the regulation ability of antioxidant enzymes, it may impair lipids, proteins, carbohydrates and nucleic acids and cause oxidative damage to cells, leading to a pathological state [15–17]. When the ocular surface tissue is subjected to environmental stress (such as ultraviolet rays, high temperature, wind and sand, etc.), the ROS level will grow sharply, and the balance between ROS and antioxidant enzymes will be disrupted [4, 18]. Subsequently, pathological changes will appear in the cornea, conjunctiva and lacrimal gland, followed by tear film hyperosmolarity and decreased stability, inducing the occurrence of DED [19, 20]. Therefore, developing new therapies that possess anti-oxidative and anti-inflammatory capabilities by attenuating excessive ROS on the ocular surface is highly desirable in DED treatment [21].

Nanomaterials with enzyme-mimicking activity have been widely used in biomedical applications [22–24]. Cerium oxide nanozyme is one of the most reported nanomaterials with enzyme-mimicking activity. Since electrons can shuttle between +3 (reduced, electron donor) and +4 (oxidized, electron acceptor), resulting in valence conversion of cerium atoms [25, 26]. Therefore, cerium oxide nanozymes have the capability of simulating ROS scavenging properties of superoxide dismutase (SOD) and catalase (CAT) [27–29]. SOD could catalyze the dismutation of $O_{2}^{-\cdot }$ to produce H_2_O_2_ and O_2_. CAT could further catalyze H_2_O_2_ to produce H_2_O and O_2_. This inherent property of cerium oxide nanozymes enables them to act as excellent ROS scavengers to protect cells from ROS damage [30–32]. Cerium oxide nanozymes have been identified as promising candidates for curing oxidative stress-related diseases [33–35]. Since their aqueous insolubility restricts plausible therapeutic effects, most current efforts have focused on improving their solubility and stability in water [35, 36]. Several studies have reported the wide applications of cerium oxide nanozymes in multi-system antioxidant therapy. For example, Zeng *et al.* [37] developed a ceria oxide nanozymes-loaded hyaluronic acid nanovesicle to relieve the hypoxic tumor microenvironments and achieve targeted delivery of photosensitizers. Mitra *et al.* [38] combined cerium oxide nanozyme and hyaluronic acid to reduce ROS-induced pro-angiogenic vascular endothelial growth factor expression and inhibit laser-induced choroidal neovascularization through scavenging overexpressed intracellular ROS, thus achieving effective treatment of age-related macular degeneration.

Polymeric materials have been widely used as delivery carriers for ophthalmic treatment [39, 40]. Branched poly(ethylene imine)-graft-poly(ethylene glycol) (bPEI-g-PEG), made from polyethyleneimine (PEI) and poly(ethylene glycol) (PEG), is a branched and positively charged copolymer [41]. PEI contains a multitude of primary, secondary and tertiary amines and other groups, which can be combined with cerium ions through coordination bonds [42]. PEG, which is highly hydrophilic and flexible, can reduce the cytotoxicity of the bPEI-g-PEG copolymer [43]. Functionalization of cerium oxide nanozymes with hydrophilic polymers with positively charged groups enhances in effect the dispersity and stability of the composite nanoparticles through electrostatic repulsion. In addition, the cellular uptake of nanozymes can be remarkably enhanced by cationic modification. Simultaneously, nanozymes are efficiently delivered to relevant sites due to high membrane-binding affinity. Therefore, we are interested in developing water soluble and highly biocompatible cerium oxide nanozymes by introducing stable hydrophilic chains on the surface. PEIs containing a large number of amino groups have been shown to have low toxicity and good biocompatibility for conjugation with ceria nanozymes [44]. Moreover, the highly hydrophilic PEG can further improve the biocompatibility of PEI. Hence, it is of interest to achieve the combination of cerium oxide nanozymes and bPEI-g-PEG to obtain water-soluble, stable, and biocompatible nanoformulations with enhanced antioxidant properties.

**Figure 1.**
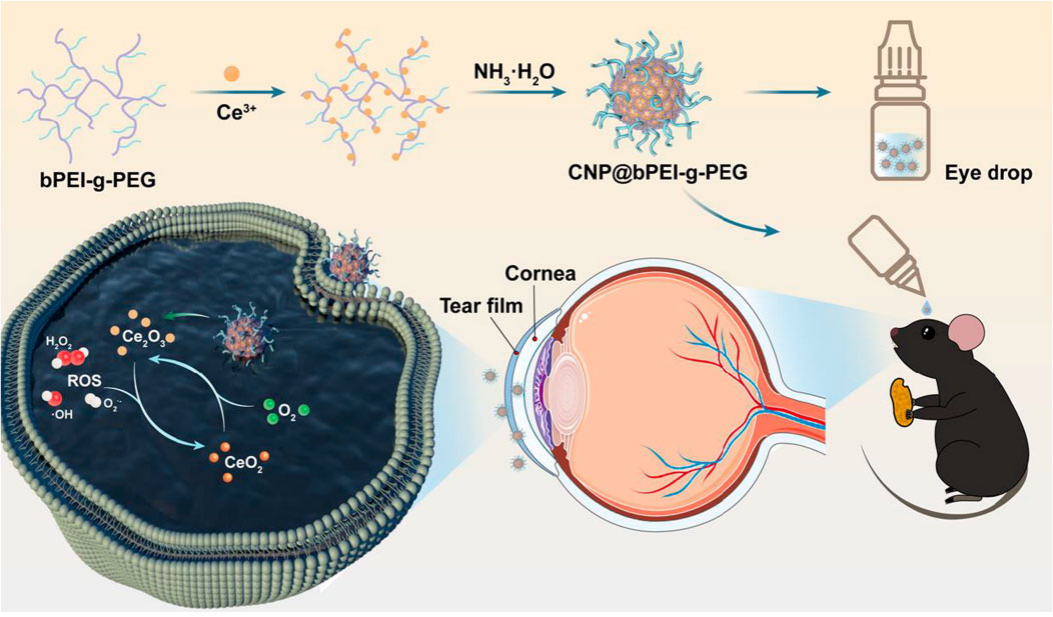
Schematic illustration of the CNP@bPEI-g-PEG nanozymes and antioxidative damage mechanism for DED.

In this study, a cerium oxide nanozyme modified with bPEI-g-PEG copolymer (referred to as CNP@bPEI-g-PEG) was successfully fabricated. As illustrated in Fig. 1, we envision that the positively charged nanozymes could increase their residence time on the ocular surface, scavenge overexpressed ROS and alleviate oxidative damage and inflammatory response, thus achieving effective treatment of DED.

## Materials and methods

### Materials

Cerium chloride heptahydrate (CeCl_3_·7H_2_O), bPEI-g-PEG and fluorescein isothiocyanate (FITC) were purchased from Sigma-Aldrich (USA). DMEM/F12 medium, 0.05% Trypsin-EDTA (1×) were purchased from Gibco Life Technologies (Grand Island, NY, USA). Fetal bovine serum was obtained from Invitrogen (Carlsbad, CA, USA). Scopolamine hydrobromide was purchased from Adamas-beta (China). About 0.3% sodium hyaluronate eye drops were purchased from Santen Pharmaceutical Co., Ltd. (China). The human corneal epithelial cells (HCECs) were purchased from ATCC (USA).

### Preparation and physicochemical characterization of CNP@bPEI-g-PEG nanozymes

To prepare CNP@bPEI-g-PEG nanozymes, 0.2 ml of 0.7 M CeCl_3_·7H_2_O solution was added into a 5-ml solution of 2% (w/v) bPEI-g-PEG under continual stirring for 20 min at room temperature to receive a transparent mixture. About 0.2 ml of ammonia was added dropwise to the mixture at a uniform rate. The reaction was continued to stir at room temperature for 12 h. After dialysis (Thermo Fisher Scientific, 14 kDa MWCO cutoff) against distilled water for 2 days, the mixture solution was neutralized to physiological pH 7.4 using acetic acid and lyophilized. The CNP suspension was prepared in parallel to CNP@bPEI-g-PEG following the same methodology. After preparation, the CNP@bPEI-g-PEG nanozymes were characterized by several scientific techniques. The morphology and structure of the nanozymes were observed by transmission electron microscopy (TEM, JEM2100F, JEOL, JPN). High-resolution TEM (HR-TEM) technique was applied to visualize the size and exact shape of CNP@bPEI-g-PEG. The characteristic absorption peaks of CNP@bPEI-g-PEG and CNP were characterized by UV-vis spectroscopy (UV-Vis, UV-1780, Shimadzu, JPN, respectively). Thermogravimetric analysis (TGA, STA 449F3, NETZSCH, GER) was utilized to quantitatively determine the content of bPEI-g-PEG in the CNP@bPEI-g-PEG nanozymes. The hydrodynamic diameters and zeta potentials of CNP@bPEI-g-PEG and CNP were measured by dynamic light scattering (DLS, Zetasizer Nano ZS ZEN3600, Malvern, UK). The presence of Ce^3+^/Ce^4+^ was determined by X-ray photoelectron spectroscopy (XPS, ESCALAB 250Xi, Thermo-Fisher Scientific, USA).

### SOD-like, CAT-like activity and regeneration capacity of CNP@bPEI-g-PEG nanozymes

SOD can catalyze the disproportionation of superoxide anion radical ($O_{2}^{\cdot -}$) to generate oxygen and hydrogen peroxide (H_2_O_2_), and CAT can produce oxygen by catalyzing H_2_O_2_, both of which play a crucial role in retaining the balance of oxidants and antioxidants *in vivo*. The enzymatic activities of CNP@bPEI-g-PEG and CNP were detected according to the instructions of SOD and CAT assay kits (Beyotime Biotechnology, China). In this regard, high (10 mg/ml) and low (1 mg/ml) concentrations of CNP@bPEI-g-PEG were utilized and CNP was used as the control. To evaluate the regenerable properties of CNP@bPEI-g-PEG nanozymes, 1 M H_2_O_2_ was added to CNP@bPEI-g-PEG and CNP solutions on days 0, 7, and 10. The samples were stored at 25°C after recording.

### *In vitro* biocompatibility of CNP@bPEI-g-PEG nanozymes

#### Cell viability

The cell counting kit-8 kit (CCK-8, Beyotime Biotechnology, China) was used to detect the cytotoxicity of bPEI-g-PEG and CNP@bPEI-g-PEG. HCECs were seeded into 96-well plates in 100 μl at a density of 1 × 10^4^ cells/well and incubated overnight in a 37°C, 5% CO_2_ cell culture incubator for 24 h. Then, the original culture media in each well were replaced with 100 μl of fresh media containing different concentrations (0.625, 1.25, 2.5, 5, 10, 20 μg/ml) of bPEI-g-PEG or CNP@bPEI-g-PEG. The plates were continuously incubated for 24 h. Thereafter, the drug-containing medium in the well plate was eliminated, and then the plate was washed once with PBS. CCK-8 was diluted with a complete medium to a concentration of 10% working solution. Then, each well was added with 100 μl of CCK-8 working solution and incubated for another 2 h in a humidified atmosphere with 5% CO_2_. The absorbance at 450 nm was recorded using a full-wavelength microplate reader (SpectraMax M5, Thermo-Fisher Scientific, USA). The cell viability was evaluated according to the instructions.

#### Live and dead cell assay

HCECs were seeded into a 96-well plate at a density of 5 × 10^3^ per well in a 37°C, 5% CO_2_ incubator. After the cells were allowed to attach for 24 h, the original medium was replaced by a complete medium containing different concentrations of CNP@bPEI-g-PEG nanozymes (0.625, 1.25, 2.5, 5, 10, 20 μg/ml). After co-incubating, the plate was gently washed once with sterile PBS solution. The cells were simultaneously fluorescently stained using the Calcein/PI cell viability/cytotoxicity assay kit (Beyotime Biotechnology, China) and incubated in dark for 30 min. The cells were then observed and photographed under an inverted fluorescence microscope (DMi8, Leica, GER).

### *In vivo* biocompatibility of CNP@bPEI-g-PEG nanozymes

To evaluate the biocompatibility of CNP@bPEI-g-PEG nanozymes *in vivo*, the mice were divided at random into three groups: (i) control group; (ii) bPEI-g-PEG group; (iii) CNP@bPEI-g-PEG group. PBS, bPEI-g-PEG and CNP@bPEI-g-PEG were instilled into the mice’s eyes three times a day for 7 days. On days 0 and 7, slit lamp examination was performed to evaluate corneal fluorescein staining, corneal edema, corneal neovascularization, and corneal and conjunctival inflammation. The mice were euthanized after the final assessment and then the eyeballs were enucleated and fixed in 4% paraformaldehyde (PFA) overnight at 4°C. After dehydration, the specimens were embedded in paraffin. Histological sections of tissues (cornea, iris and retina) were stained with HE staining and observed using an upright fluorescence microscope (DM4B, Leica, GER).

### Determination of intracellular ROS scavenging

To explore the ability of CNP@bPEI-g-PEG nanozymes to scavenge intracellular ROS, H_2_O_2_ was used to simulate excessive ROS. First of all, the appropriate H_2_O_2_ stimulation concentration was chosen. After confirming that the cells were confluent to the bottom of the well plate, different concentrations of hydrogen peroxide were added and co-incubated with HCEC for 2 h. Cell viability was quantitatively detected by CCK-8 after 24 h. A total of 100 μl of H_2_O_2_ (600 μM) of fresh medium was added to each well to stimulate HCECs at 37°C for 2 h. The medium containing H_2_O_2_ was discarded and the 96-well plate was washed with PBS once. Then, 100 μl of complete medium containing different concentrations of CNP@bPEI-g-PEG (0, 5, 10 μg/ml) was added and co-incubated for 24 h, with five parallels per group. The subsequent steps are the same as the above cytotoxicity test. Another group without H_2_O_2_ stimulation (Control group) was set up as the normal control.

DCFH-DA, a ROS detection fluorescent probe is generally used as a sensitive marker of cellular oxidation processes since intracellular ROS can oxidize non-fluorescent DCFH to generate fluorescent DCF [45]. Briefly, HCECs were incubated with CNP@bPEI-g-PEG nanozymes for 24 h. Subsequently, DCFH-DA was diluted with serum-free media to a final concentration of 10 μM according to the instruction of the ROS assay kit (Beyotime Biotechnology, China). After being washed once with sterile PBS, HCECs were mixed with DCFH-DA, co-incubated for 20 min and rinsed with serum-free cell culture medium twice to sufficiently remove excessive DCFH-DA. A total of 600 μM H_2_O_2_ was added and co-incubated for additional 2 h at 37°C. The DCF fluorescence was recorded in the FITC channel of a fluorescence microscope (DMi8, Leica, GER) using the same exposure time. The HCECs untreated with H_2_O_2_ were set as the control group while HCECs treated with H_2_O_2_ but not incubated with the drug were set as the model group.

### *In vitro* cellular uptake of CNP@bPEI-g-PEG nanozymes

To evaluate the cellular uptake capability of CNP@bPEI-g-PEG nanozymes by HCECs, CNP@bPEI-g-PEG was labeled with FITC. Briefly, 0.875 ml of CNP@bPEI-g-PEG (10 mg/ml) was mixed with 0.125 ml of FITC (3.6 mg/ml) at room temperature overnight. The unreacted FITC molecules were removed by dialysis (MWCO = 1000) against distilled water. HCECs were treated with FITC-labeled CNP@bPEI-g-PEG for 24 h and then fixed with 4% PFA for 30 min. After washing with PBS three times, the cells were stained with red fluorescent probe (Dil, Beyotime Biotechnology, China) for 3 min, and mounted with a DAPI-containing anti-fluorescence quencher. Confocal laser microscopy (two-photon + super-resolution) (LSM 880, Zeiss, GER) was used to observe the distribution of the nanozymes in cells.

### Creation of DED mouse model

Female C57BL/6 J mice aged 6–8 weeks were purchased from Shanghai Jiesijie Laboratory Animals Co, Ltd. Care and use of the mice were supported by the Experimental Animal Ethics Committee of Wenzhou Medical University. The inclusion criteria of experimental mice were as follows: no corneal ulcers, no old leukoplakia and healthy mice with corneal fluorescein sodium staining score less than 10 points under slit lamp examination.

To build DED model, 200 μl of scopolamine hydrobromide (2.5 mg/ml) was subcutaneously injected to inhibit tear secretion four times a day (9:00 a.m., 12:00 a.m., 3:00 p.m., 6:00 p.m.) for seven consecutive days [46]. The mice were divided at random into four groups: (i) control group, normal mice with PBS; (ii) model group, DED mice with PBS; (iii) SH group, DED mice with SH (0.3% sodium hyaluronate); (iv) CNP@bPEI-g-PEG group, DED mice with CNP@bPEI-g-PEG nanozymes. A total of 5 μl eye drops were superficially administered to the ocular surface once a day for seven consecutive days.

### *In vivo* therapeutic effects of CNP@bPEI-g-PEG nanozymes

Corneal fluorescein staining: 5 μl of sodium fluorescein solution was instilled into the conjunctival sac of the mice, and the eyes were manually assisted to blink 2–3 times. After 2 min, the sodium fluorescein staining of the mouse cornea was observed using a slit lamp microscope under cobalt blue light. Then, the overall corneal damage was scored and recorded by an experienced ophthalmologist. Briefly, the cornea was first divided into five regions: superior, inferior, nasal, temporal and central, and then scored. According to the standard National Eye Institute scoring system [47], the level of corneal fluorescein staining was determined to be 0 (no punctate staining), 1 (slight punctate staining), 2 (diffuse punctuate staining) or 3 (severe patchy staining). The total scores of the five regions were summed as the final corneal fluorescein staining scores.

Probability of not recovered: The severity of the dry eye state was assessed using the probability of not recovered. It was regarded as a recovery state when the corneal fluorescein sodium staining score decreased by four points or more. Recovery status was defined as 1, and not recovered was 0. The results were shown as survival curves.

Histological evaluation: On day 7, mice were humanely sacrificed after slit lamp assessment. The eyeballs with integral conjunctiva were quickly removed, immediately rinsed in 0.9% saline and fixed in 4% PFA. The specimens were dehydrated and embedded in paraffin. After cooling and solidification, the specimens were cut into 5 μm-thick slices, flattened and transferred to glass slides. HE staining was performed after drying. The morphology of corneal, conjunctival epithelium and goblet cells were observed using an upright fluorescence microscope (DM4B, Leica, GER).

### Statistical analysis

All data were expressed as mean ± SD. The statistical differences among groups were determined using Student’s t test or one-way ANOVA. A *P* values of less than 0.05 was considered statistically significant (*P < 0.05, **P < 0.01, ***P < 0.001).

## Results and discussion

### Physicochemical characterization

**Figure 2.**
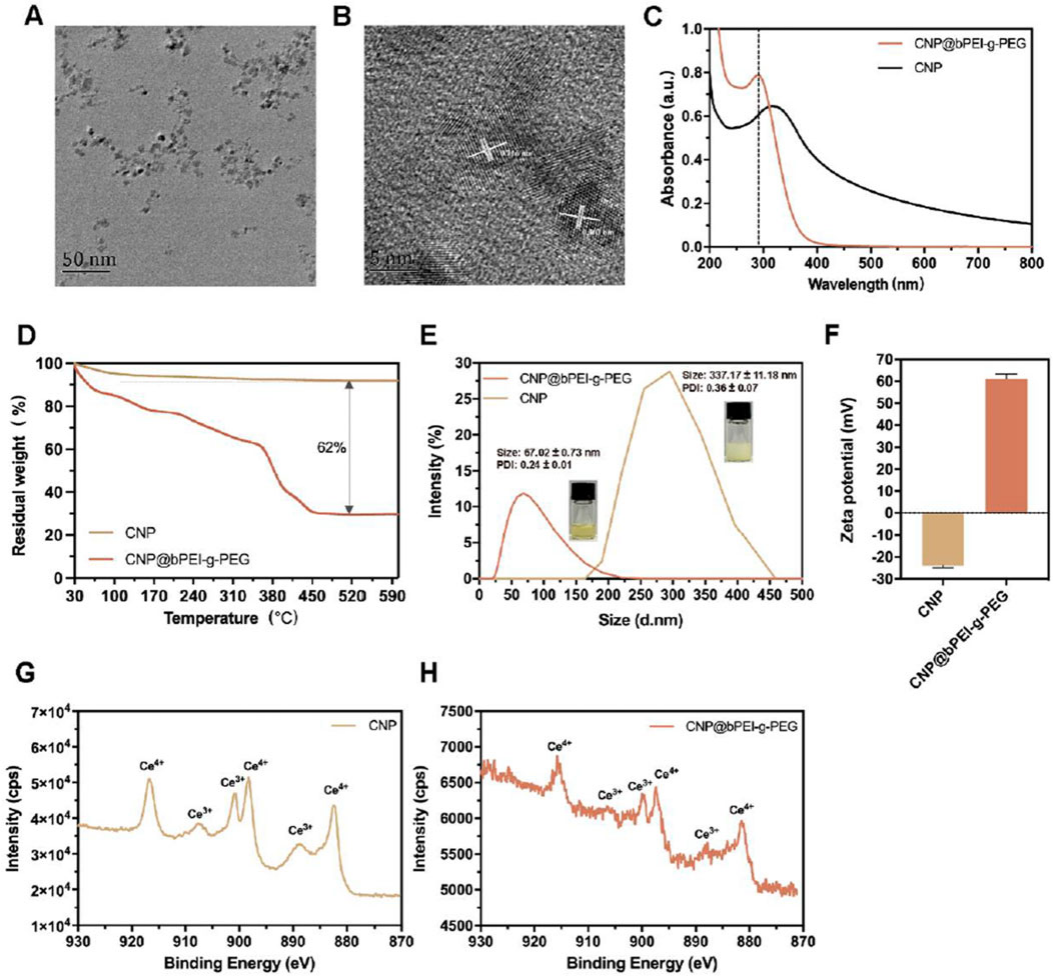
Characterization of CNP@bPEI-g-PEG. (**A**) TEM images of CNP@bPEI-g-PEG nanozymes. Scale bar = 50 nm. (**B**) HR-TEM images. Scale bar = 5 nm. (**C**) UV-Vis spectrograms of CNP and CNP@bPEI-g-PEG. (**D**) TGA curves of CNP and CNP@bPEI-g-PEG. (**E**) Size, PDI and representative histogram of size distributions of CNP and CNP@bPEI-g-PEG. (**F**) Zeta potential of CNP and CNP@bPEI-g-PEG. (**G, H**) XPS test result indicating the chemical valence of CNP and CNP@bPEI-g-PEG.

In this study, CNP@bPEI-g-PEG nanozymes were prepared using the NH_3_·H_2_O precipitation method. The synthetic pathways and reaction modes are presented in Fig. 1. TEM images in Fig. 2A revealed that the CNP@bPEI-g-PEG nanozymes were particles with a diameter of 5–10 nm. The representative HR-TEM images of CNP@bPEI-g-PEG (Fig. 2B) showed the crystalline nature and lattice constant of 0.303–0.316 nm. UV-Vis spectra of CNP and CNP@bPEI-g-PEG in Fig. 2C indicated that a blue shift of the characteristic absorption peaks from 320 to 290 nm appeared to a certain extent after CNP was combined with bPEI-g-PEG through coordination bonds. Figure 2D showed the TGA curve of CNP and CNP@bPEI-g-PEG in the range of 30–600°C and under an air atmosphere. The TGA curve of CNP@bPEI-g-PEG performed a continuous weight loss (100–30%) within the range of 30–460°C due to a decomposition of the adsorbed water and bPEI-g-PEG. The left 30% was attributed to the inorganic cerium content of the CNP@bPEI-g-PEG. This result demonstrated that bPEI-g-PEG and naked cerium oxide nanoparticles combined successfully with the former constituting up to 62 wt%.

As demonstrated in Fig. 2E, the hydrodynamic diameters of CNP and CNP@bPEI-g-PEG were determined by DLS measurement to be 337.17 ± 11.18 and 67.02 ± 0.73 nm, respectively. After being modified by bPEI-g-PEG, the CNP@bPEI-g-PEG nanozymes’ PDI declined from 0.36 ± 0.07 to 0.24 ± 0.01. The representative nanoparticle size distribution histograms of CNP and CNP@bPEI-g-PEG were presented in Fig. 2E, simultaneously. The zeta potential of CNP@bPEI-g-PEG (+61.07 ± 2.23 mV, Fig. 2F) was also significantly (***P < 0.001) higher than CNP (−23.90 ± 1.05 mV). The higher particle size value of CNP may be due to the agglomeration or aggregation into clusters, which may account for its lower solubility and stability in the solution. The faint yellow color of the CNP@bPEI-g-PEG solution (the inset of Fig. 2E) illustrated the presence of the Ce^3+/4+^ valence states at the same time. XPS analysis showed that CNP@bPEI-g-PEG (Fig. 2H) had the same Ce^3+/^Ce^4+^ (oxidation/reduction) pattern as CNP (Fig. 2G), thus confirming the presence of both oxidation states in CNP@bPEI-g-PEG.

### Regenerable SOD-like and CAT-like nanozyme activity

**Figure 3.**
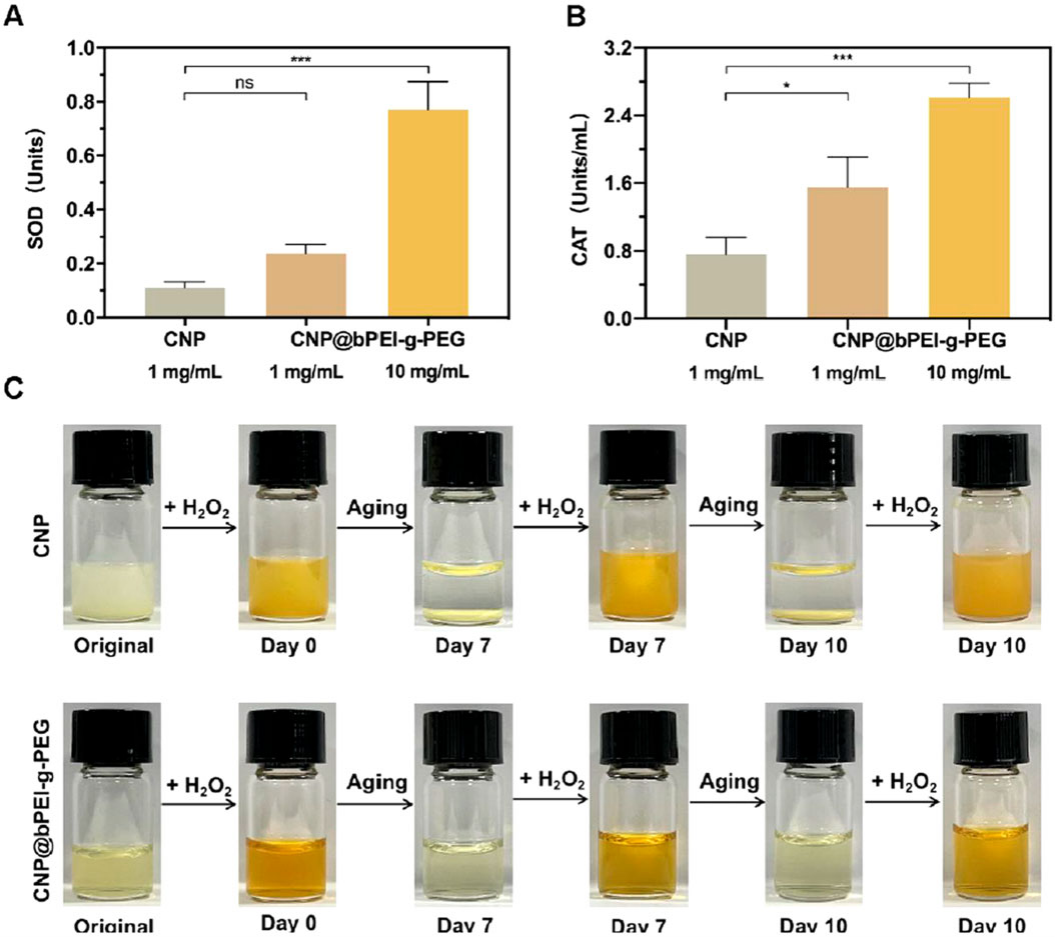
SOD-like and CAT-like activities and regenerative properties of CNP and CNP@bPEI-g-PEG. (**A, B**) Detection of SOD and CAT mimicking function of CNP and CNP@bPEI-g-PEG. (**C**) Images of color changes of CNP (top) and CNP@bPEI-g-PEG (bottom) on addition of H_2_O_2_.

Due to its capability of creating oxygen vacancies in the crystal lattice, cerium oxide nanoparticles possess exceptional redox properties. When faced with over-oxidative stimulation, cerium oxide nanoparticles could mimic SOD and CAT activities to scavenge excessive ROS. Cerium oxide nanoparticles with small particle sizes are more advantageous in exerting their enzymatic activities. As indicated in Fig. 3A, the high concentration group of CNP@bPEI-g-PEG exhibited greater SOD-like activity than the low concentration of CNP@bPEI-g-PEG and CNP group (***P < 0.001). Compared with CNP, the CAT-mimicking activity of CNP@bPEI-g-PEG was enhanced as its concentration increased (Fig. 3B). Significant differences all can be found in the 1 mg/ml (*P < 0.05) and the 10 mg/ml (***P < 0.001) CNP@bPEI-g-PEG by comparison with CNP group. In Fig. 3C, the color of the CNP and CNP@bPEI-g-PEG solutions rapidly turned to dark yellow (day 0) upon the addition of 1 M H_2_O_2_. The solutions were reinstated to their original state on day 7. Thereafter, we added H_2_O_2_ caused the colors to change to dark yellow again, the same phenomenon was observed on day 10. Therefore, these results indicated that the pale-yellow color of CNP@bPEI-g-PEG was due to the coexistence of Ce^3+^ (colorless) and Ce^4+^ (pale yellow) oxides.

In the present study, CNP@bPEI-g-PEG nanozymes were confirmed to have SOD and CAT double enzyme activities, simultaneously. The coexistence of Ce^3+^/Ce^4+^ ensured the realization of the redox cycle between Ce^3+^ and Ce^4+^ in our nanozymes. Above all, CNP@bPEI-g-PEG nanozymes had an excellent role in terms of regenerative properties and powerful antioxidant functions.

### *In vitro* biocompatibility of CNP@bPEI-g-PEG nanozymes

**Figure 4.**
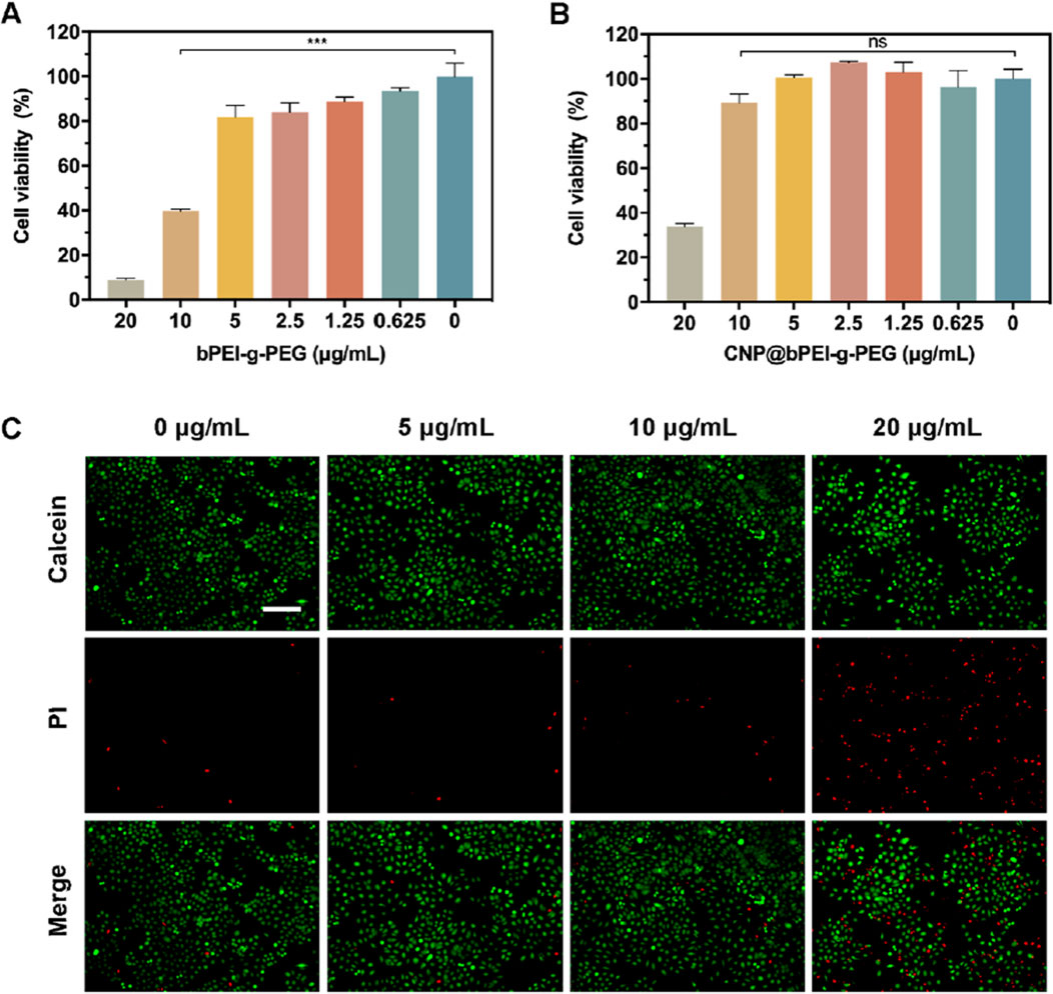
(**A, B**) Toxicity of different concentrations of bPEI-g-PEG and CNP@bPEI-g-PEG to HCEC incubated for 24 h. (**C**) Fluorescence image of calcein/PI staining. Scale bar = 200 µm.

Given the prominent capability to attenuate ROS, the current application of cerium oxide nanozymes in biomedicine to treat oxidative injury diseases has a broad and bright future for clinical translation. Therefore, it is necessary to evaluate the biosafety of CNP@bPEI-g-PEG nanozymes. First, the cytotoxicity of different concentrations of bPEI-g-PEG and CNP@bPEI-g-PEG on HCECs was investigated by CCK-8 assay. As illustrated in Fig. 4A, bPEI-g-PEG at concentrations less than 0.625 µg/ml was well tolerated while significant cytotoxicity was observed with the concentration up to 10 µg/ml. In Fig. 4B, treatment with CNP@bPEI-g-PEG at the indicated concentrations (0.625, 1.25, 2.5, 5, 10 µg/ml) had no significant influence (*P* > 0.05) on HCECs viability comparing with the untreated group (0 µg/ml), but cell viability was significantly reduced with the concentration up to 20 µg/ml. Hence, the overall results revealed that CNP@bPEI-g-PEG-treated cells are conclusively less toxic than CNP. This phenomenon may be due to the toxic PEI binds with cerium atoms through coordination bonds, and then self-entangles to form an inner nucleus. At the same time, the long PEG chains which can enhance biocompatibility assemble with them into spheres externally.

The morphological changes and cell density of HCECs treated with different concentrations of CNP@bPEI-g-PEG were further observed by live/dead cell staining. Calcein localizes live cells and exhibits green fluorescence while PI localizes dead cells and exhibits red fluorescence. As shown in Fig. 4C, compared with the control group (0 µg/ml), the cell morphology did not change obviously after co-incubation with different concentrations of CNP@bPEI-g-PEG. When the concentration was 5 and 10 µg/ml, cell pseudopodia were distinct, and few dead cells were observed. Consistent with the results of CCK-8, a high concentration of 20 µg/ml reduced the number of viable cells and increased that of dead cells. These data, taken together, revealed that CNP@bPEI-g-PEG nanozymes had high biocompatibility without obvious cytotoxicity. It indicated that CNP@bPEI-g-PEG nanozymes had acquired the capacity to act as a safe nanotherapeutic method for future clinical applications.

### *In vivo* biocompatibility of CNP@bPEI-g-PEG nanozymes

**Figure 5.**
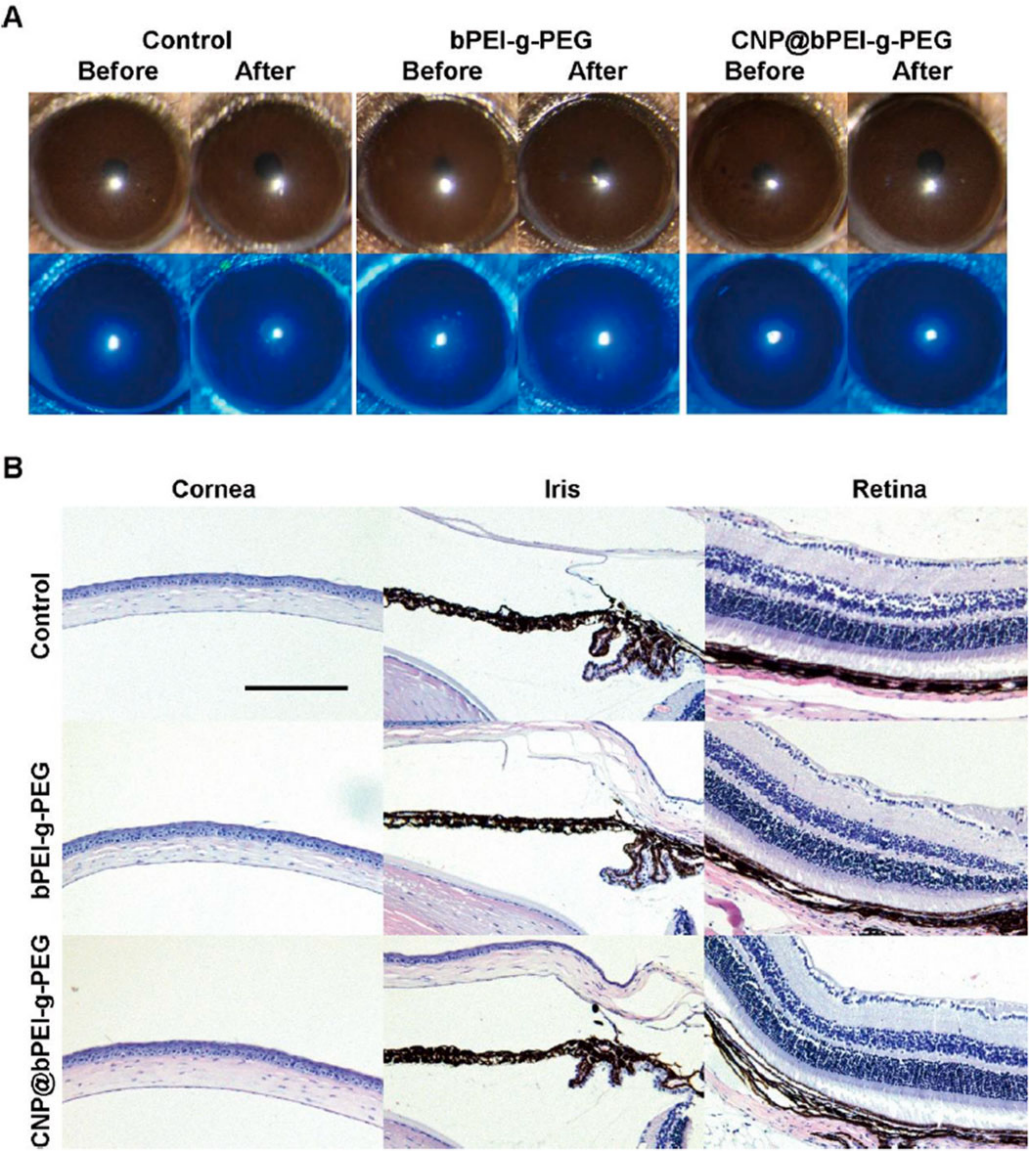
(**A**) Corneal fluorescein sodium staining images before and after drug instillation. (**B**) Histopathology microscopy of ocular tissues, including cornea, iris and retina. Scale bar = 200 µm.

To intuitively examine the biocompatibility of CNP@bPEI-g-PEG nanozymes *in vivo*, the silt lamp assessment and ocular histocompatibility evaluation were applied. After the drug was instilled (Fig. 5A), the ocular surface of the mice was smooth, with no corneal edema, neovascularization and opacity, no corneal and conjunctival inflammation, and no obvious fluorescein sodium staining under cobalt blue light, which was no significant difference from before the drug instillation. As shown in Fig. 5B, histocompatibility detection was performed by viewing HE-stained slides to assess the structure and integrity of tissues (cornea, iris and retina). Compared with the normal mice in the control group, the corneal surface epithelial cells in the bPEI-g-PEG and CNP@bPEI-g-PEG groups had normal morphology and cell arrangement, and the iris and retina were intact. The cornea, iris and retina had no inflammatory changes, no inflammatory cell infiltration, and no significant pathological changes, indicating that CNP@bPEI-g-PEG nanozymes did not cause ocular inflammation and had good histocompatibility.

### Determination of intracellular ROS

Numerous studies have established that oxidative stress plays a vital role in the pathogenesis of DED. Under physiological conditions, ROS are in equilibrium with antioxidant enzymes on the ocular surface. However, ROS may lead to oxidative stress and damage to the cornea and conjunctiva due to overexpression or poor endogenous defense systems, thereby inducing death signaling pathways to produce severe pathological conditions, such as DED. Pathological levels of ROS can cause the progress of DED through oxidative damage. Furthermore, previous studies demonstrated the protective effect of antioxidants on ocular tissues by eliminating overexpressed ROS [11]. Therefore, the urgent priority is evaluating the ROS scavenging effect of CNP@bPEI-g-PEG nanozymes.

**Figure 6.**
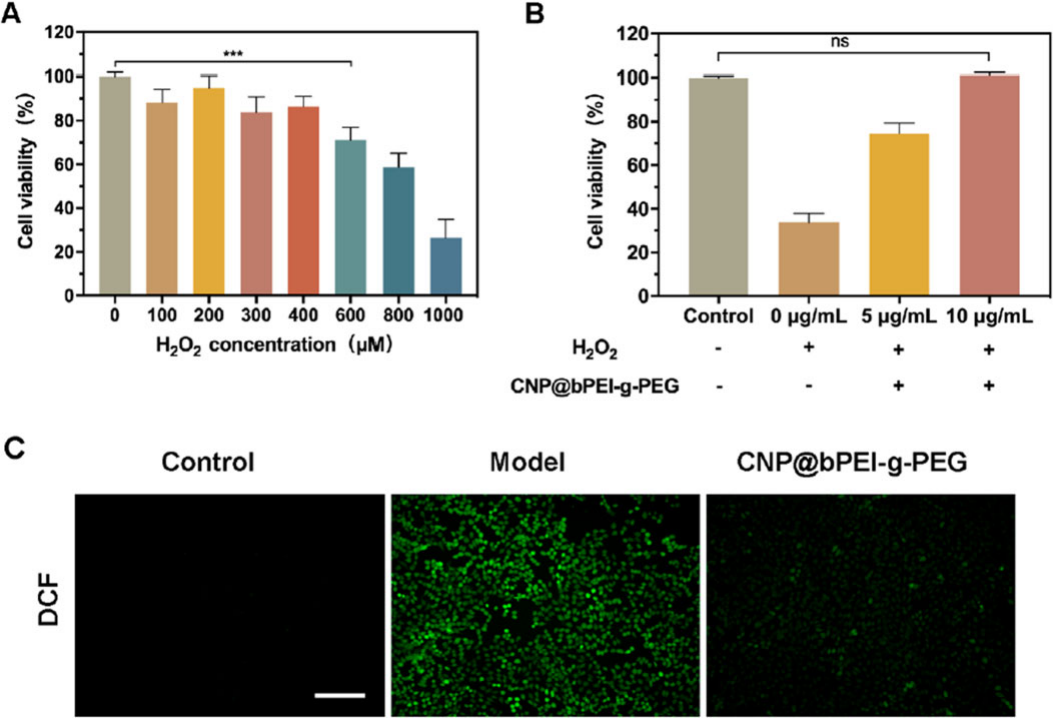
(**A**) Quantitative cell viability of HCECs after stimulating by H_2_O_2_ in different concentrations. (**B**) Quantitative cell viability of HCECs with CNP@bPEI-g-PEG in different concentrations after stimulating by H_2_O_2_ (600 µM). (**C**) DCF fluorescence images of HCECs after exposure to H_2_O_2_ and then treated with CNP@bPEI-g-PEG. The HCECs untreated with H_2_O_2_ were set as the Control group, while HCECs treated with H_2_O_2_ but not incubated with the drug were set as the model group. Scale bar = 200 µm.

By stimulating HCECs with different concentrations of H_2_O_2_ and detecting their cell viability, the optimal H_2_O_2_ stimulation concentration can be determined. As presented in Fig. 6A, cell viability decreased when treated with hydrogen peroxide. When H_2_O_2_ concentration ranged from 100 to 400 μM, the viability of HCECs fluctuated slightly, but no significant decrease in cell viability was observed compared to the control group (0 μM). With the continuous increase of H_2_O_2_ concentration, the cell viability decreased drastically. Cell viability of less than 80% was observed when treated with H_2_O_2_ at the concentration of 600 μM while treatment with higher concentration of H_2_O_2_ (1 mM) led to only about 30% of cell viability. Thus, the optimal concentration of H_2_O_2_ was determined to be 600 μM. The significant decline in cell viability can be attributable to the destruction of cell membranes by high concentrations of H_2_O_2_. As shown In Fig. 6B, with the increase of CNP@bPEI-g-PEG, the cell viability increased continuously. When the concentration of CNP@bPEI-g-PEG was 0 µg/ml, the cell viability went down significantly (**P < 0.01), indicating that H_2_O_2_ caused a damaging effect on HCECs, which induced the cell over-oxidative model caused by excessive ROS. When concentration of CNP@bPEI-g-PEG drug was 10 µg/ml, no significant difference (*P* > 0.05) in cell viability was observed compared with the control group that was not stimulated by H_2_O_2_.

The fluorescence intensity of DCF was adopted as an indication of the content of ROS. As shown in Fig. 6C, almost no green fluorescence was observed in the control group while the model group exhibited a dramatic amount of fluorescence. The DCF fluorescence of the CNP@bPEI-g-PEG group was significantly decreased, which can be attributed to the inhibitory effect of CNP@bPEI-g-PEG on ROS. Taken together, these results clearly demonstrated that CNP@bPEI-g-PEG nanozymes could drastically scavenge intracellular ROS within HCECs. This provided strong support for the application of CNP@bPEI-g-PEG nanozymes in oxidative injury therapy in biomedicine.

### *In vitro* cellular uptake

**Figure 7.**
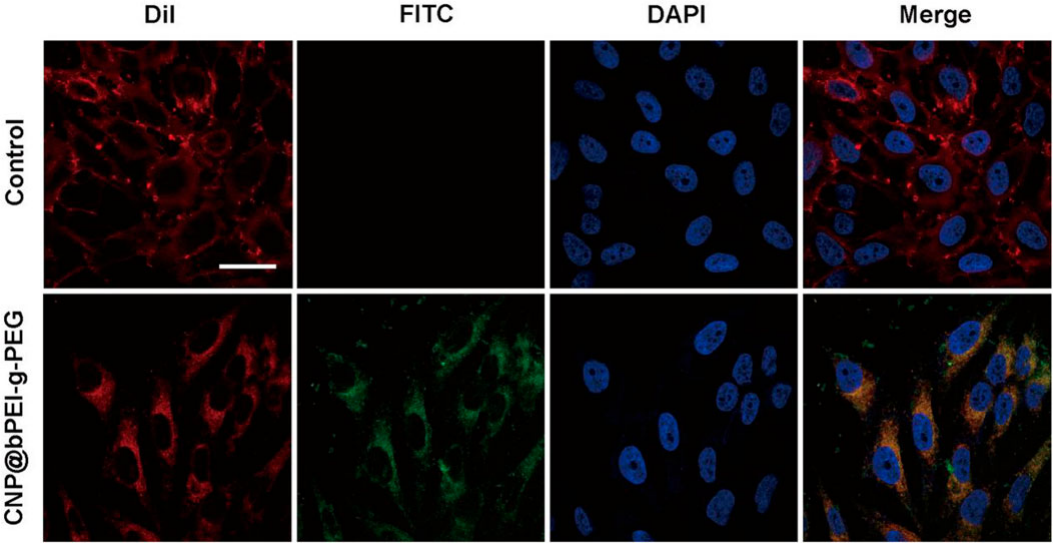
The cellular uptake of FITC-labeled CNP@bPEI-g-PEG. Scale bar = 30 µm.

Co-incubation of FITC-labeled CNP@bPEI-g-PEG with HCECs and observing its distribution within HCECs were conducted to determine the cellular uptake capability. As shown in Fig. 7, the morphology of cellular membranes in both groups remained intact, and pseudopodia were visible; the nuclei were round or oval with abundant nucleoplasm and complete structure, suggesting a good growth state. The difference was in the FITC channel; HCECs incubated with CNP@bPEI-g-PEG-FITC showed green fluorescence, and the fluorescence was located inside the cell membrane and outside the nucleus. In contrast, no green fluorescence distribution can be seen in the control group, which indicated that CNP@bPEI-g-PEG nanozymes were endocytosed into the cytoplasm. As is known to all, mitochondria are the major sites of ROS production and are distributed throughout the cytoplasm. Hence, CNP@bPEI-g-PEG nanozymes can deliver cerium oxide to the optimal location, attenuating and clearing the excess ROS produced by mitochondria. The experimental result demonstrated that the ability of HCECs to uptake CNP@bPEI-g-PEG was outstanding, and our nanozymes would reside in preferred intracellular locations to scavenge ROS.

### *In vivo* therapeutic effects

**Figure 8.**
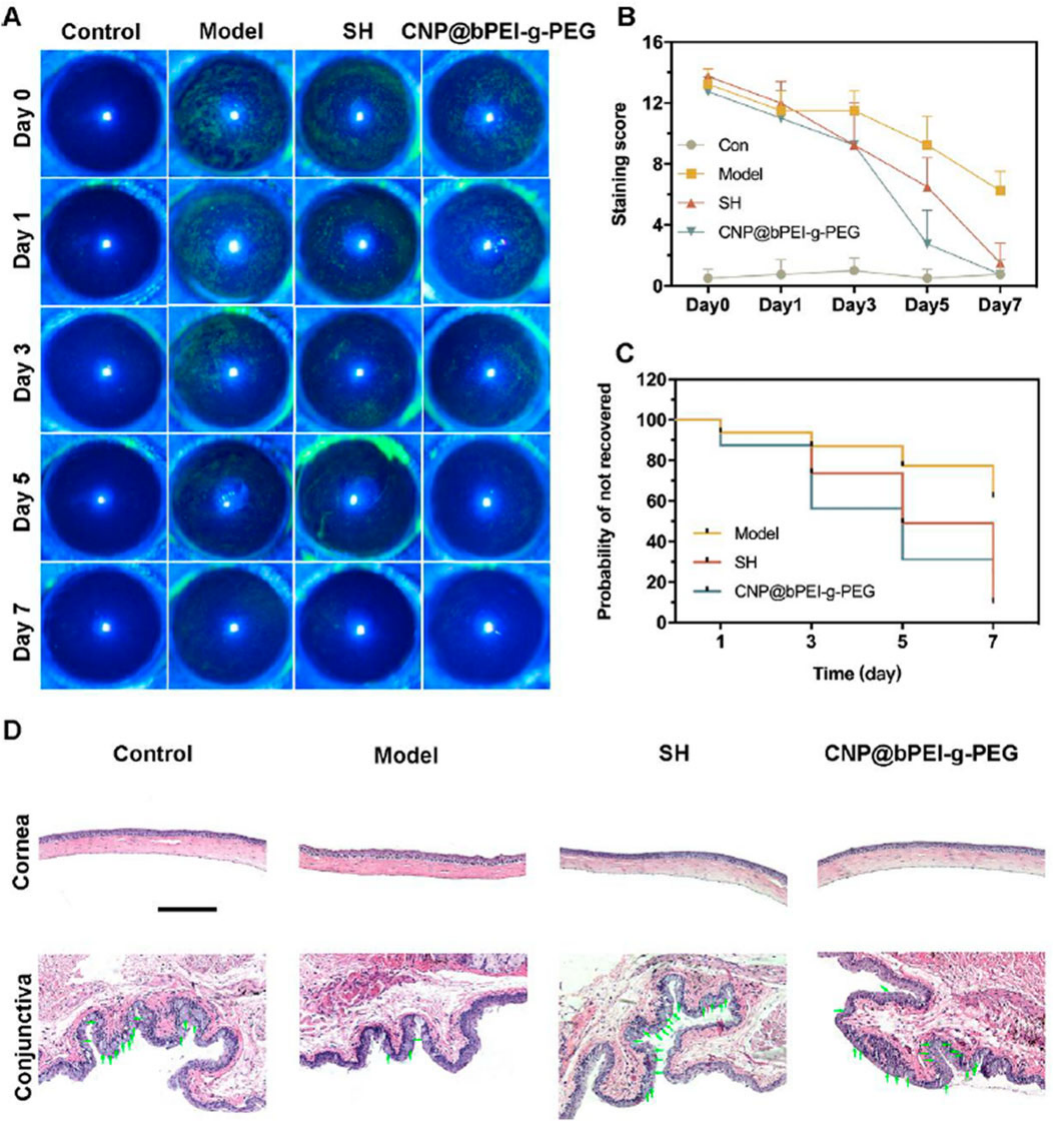
(**A**) Illustrations of the representative fluorescein staining images. (**B**) Corneal fluorescein staining scores. (**C**) Probability of not recovered. (**D**) The corneal morphology and goblet cells in the conjunctiva. Scale bar = 200 µm.

The DED therapeutic effect of the modified nanozymes by scavenging ROS was evaluated in a scopolamine-induced dry eye mouse model. It was expected that CNP@bPEI-g-PEG nanozymes could alleviate the burden of accumulated oxidative damage and their subsequent inflammatory responses. Corneal fluorescein sodium staining was performed to evaluate corneal epithelial damage, which is an important basis for the diagnosis of DED [9]. If the corneal epithelium is defective, green fluorescent spots can be seen under cobalt blue light from a slit lamp. On days 0, 1, 3, 5 and 7, corneal fluorescein sodium staining and slit lamp photography were performed for all experimental mice. Representative images were shown in Fig. 8A. The corneal epithelium of the control group was smooth, and no obvious corneal punctate coloring spots were found on day 0. As for the other three groups, the corneal epithelium was not clear with obvious diffuse flakes and lumps of coloring, and a large area of fusion in the local area indicated the successful establishment of the eye model. With the prolongation of treatment, CNP@bPEI-g-PEG showed a better therapeutic effect than SH, which is consistent with the corneal fluorescein staining score shown in Fig. 8B. The experimental results indicated materials had a similar or even better therapeutic effect than clinical first-line drugs. The scores of the control group fluctuated slightly during a 7-day period without a statistical difference (*P* > 0.05). On day 0, the fluorescein staining scores of the model, SH and CNP@bPEI-g-PEG groups were significantly increased without statistical difference among these three groups (all above 0.05). Compared to day 0, a slight statistical difference could be found in the model group (*P < 0.05) while the SH and CNP@bPEI-g-PEG groups exhibited a huge statistical difference (**P < 0.01) on day 7. At the same time, there were statistical differences between the model group and the other three groups on day 7 (**P < 0.01). However, no statistical difference appeared among the control, SH and CNP@bPEI-g-PEG groups (*P* > 0.05).

The probability of not recovered was used to describe the severity of dry eye from another perspective, while its reduction may reveal the therapeutic effect of drugs on dry eye. In Fig. 8C, the not recovered rate of the model group remained the highest during a 7-day period, while the two other groups dwindled drastically. On day 7, compared with the model group, a significant decrease of the not recovered probability was found in the CNP@bPEI-g-PEG group (*P < 0.05), and the SH group was also lower (*P < 0.05). All the results manifested CNP@bPEI-g-PEG nanozymes could alleviate corneal epithelial damage, reduce the irrecovery probability of the DED mouse model, and could be a more effective therapy for DED compared to SH, which is commonly used in clinic DED treatment.

After the dry eye model was successfully established, the ocular surface altered obviously. The therapeutic effects of CNP@bPEI-g-PEG nanozymes were further validated by histological evaluation of the cornea and conjunctiva. Figure 8D shows that the corneal epithelium of the model group was distinctly thinner and damaged, and the surface was rough and uneven. Compared with the control group, the epithelial cell layer was irregularly arranged, and the stromal layer fibers were disordered. After 7 days of treatment with CNP@bPEI-g-PEG and SH, the damaged corneal epithelial layers began to recover, and the cornea became smooth and returned to a near-normal state with epithelial thickness and stromal morphology similar to the control group. Using CNP@bPEI-g-PEG nanozymes as eye drops showed an excellent therapeutic effect rather than the deterioration of corneal epithelial defect. Conjunctival goblet cells possess the ability to secrete mucoproteins aimed at lubrication and protection of the ocular surface. The excessive accumulation of ROS could directly contribute to the apoptosis of goblet cells, reduce mucin production, disrupt tear film composition, decrease corneal wettability, corneal epithelial damage, and inflammation progression. Therefore, conjunctival goblet cells play a vital role in tear film formation, and the reduction of goblet cells indicates the progression of DED. The number of goblet cells was significantly reduced in the model group compared to the control group. After treatment with CNP@bPEI-g-PEG and SH, goblet cells returned to their original level. Given that DED is an ocular disease associated with an inflammatory response, anti-inflammatory activity studies would be considered in the following research. All in all, CNP@bPEI-g-PEG could recover the morphology of corneal, conjunctival epithelium and the number of goblet cells. CNP@bPEI-g-PEG nanozymes had both therapeutic effects and high biocompatibility.

## Conclusions

In this study, a rationally designed cerium oxide nanozyme with excellent water solubility and powerful antioxidant properties was successfully constructed and thoroughly evaluated. The DLS results showed that the introduction of bPEI-g-PEG led to the decrease of cerium oxide particle size and positive surface charge. CNP@bPEI-g-PEG nanozymes were provided with a coexisting valence state of +3 and +4 cerium ions similar to pure CNP, which can simulate the activities of SOD and CAT, and automatically regenerate. As seen in the research, no changes in HCECs viability were observed after treatment with appropriate amounts of CNP@bPEI-g-PEG, which revealed that the modified nanozymes have high biocompatibility without obvious cytotoxicity. The combination of amino group and ceria makes its surface slightly positive surface charge, which leads to CNP@bPEI-g-PEG nanozymes being easily endocytosed by HCECs and aggregated in the cytoplasm. *In vitro* assay demonstrated that the delivery of CNP@bPEI-g-PEG suppressed the excessive ROS and attenuated apoptosis induced by oxidative damage. All *in vitro* and *in vivo* studies firmly illustrated promising and potent antioxidant properties of CNP@bPEI-g-PEG nanozymes as recognized by their ROS scavenging activity. In an animal model of dry eye, CNP@bPEI-g-PEG ameliorated corneal epithelial defects and increased goblet cell numbers. Therefore, this nanozyme could be beneficial to dry eye patients, especially in severe forms. Beyond its potential application in DED, the formulation will establish a broadly applicable approach to oxidative stress-related eye diseases such as bacterial keratitis.

## Funding

This work was financially supported by the Key Scientific and Technological Innovation Projects in Wenzhou [ZY2021002] and the Medical & Health Technology Program of Zhejiang Province [2022RC051].

*Conflicts of interest statement*. None declared.
